# Sequence Variation in *Toxoplasma gondii rop17* Gene among Strains from Different Hosts and Geographical Locations

**DOI:** 10.1155/2014/349325

**Published:** 2014-07-10

**Authors:** Nian-Zhang Zhang, Ying Xu, Si-Yang Huang, Dong-Hui Zhou, Rui-Ai Wang, Xing-Quan Zhu

**Affiliations:** ^1^State Key Laboratory of Veterinary Etiological Biology, Key Laboratory of Veterinary Parasitology of Gansu, Lanzhou Veterinary Research Institute, Chinese Academy of Agricultural Sciences, Lanzhou, Gansu 730046, China; ^2^College of Animal Science and Technology, Anhui Agricultural University, Hefei, Anhui 230036, China; ^3^College of Veterinary Medicine, South China Agricultural University, Guangzhou, Guangdong 510642, China

## Abstract

Genetic diversity of *T. gondii* is a concern of many studies, due to the biological and epidemiological diversity of this parasite. The present study examined sequence variation in rhoptry protein 17 (ROP17) gene among *T. gondii* isolates from different hosts and geographical regions. The *rop17* gene was amplified and sequenced from 10 *T. gondii* strains, and phylogenetic relationship among these *T. gondii* strains was reconstructed using maximum parsimony (MP), neighbor-joining (NJ), and maximum likelihood (ML) analyses. The partial *rop17* gene sequences were 1375 bp in length and A+T contents varied from 49.45% to 50.11% among all examined *T. gondii* strains. Sequence analysis identified 33 variable nucleotide positions (2.1%), 16 of which were identified as transitions. Phylogeny reconstruction based on *rop17* gene data revealed two major clusters which could readily distinguish Type I and Type II strains. Analyses of sequence variations in nucleotides and amino acids among these strains revealed high ratio of nonsynonymous to synonymous polymorphisms (>1), indicating that *rop17* shows signs of positive selection. This study demonstrated the existence of slightly high sequence variability in the *rop17* gene sequences among *T. gondii* strains from different hosts and geographical regions, suggesting that *rop17* gene may represent a new genetic marker for population genetic studies of *T. gondii* isolates.

## 1. Introduction

*Toxoplasma gondii* can infect all warm-blooded vertebrates, including mammals and birds [1–3]. Genetic diversity of* T. gondii* is widespread due to the biological and epidemiological diversity of this parasite.* T. gondii* isolates can be clustered into six major clades [4], and genetic diversity of* T. gondii* is especially common in South America [4]. Utilizing 11 genetic markers,* T. gondii* isolates in North America and Europe are grouped into four major clonal lineage types (I, II, III, and 12) [5, 6] using PCR-RFLP.

Rhoptry kinases are involved in mediating pathogenesis of* T. gondii *[7], and they are also master regulators that manipulate the host inflammatory responses [8, 9].* T. gondii *rhoptry protein 17 (ROP17), a member of the ROP2 subfamily [10], was predicted to have a cellular localization on the parasitophorous vacuole membrane (PVM), which may participate in the manipulation of the host signalling pathways [9]. Previous studies have shown the existence of sequence variation in some ROP genes, such as* rop7*,* rop9*,* rop13*, and* rop38* [11–14]. However, it is yet to be known whether sequence diversity exists in* rop17 *gene of* T. gondii*. The objective of the present study was to examine sequence variation in* rop17 *gene among* T. gondii *strains representing different genotypes and host and origins.

## 2. Materials and Methods

### 2.1. *T. gondii* Isolates 

Ten* T. gondii* strains collected from different hosts and locations were used for analysis in this study (Table 1). These strains have been genotyped and their genomic DNA has been prepared as described previously [15–17].

### 2.2. Amplification of* rop17* Genes and Sequencing

The* rop17* gene was amplified by PCR. Two primers were designed based on the* rop17* sequence of* T. gondii* RH strain available in GenBank (accession number: KC997178): ROP17F, 5′-AGGACAACACTAGGTAGCGAGAACC-3′, and ROP17R, 5′-TGGCGAAGTCAAGAGACGACGCAG-3′. Each reaction was performed in a total volume of 25 *μ*L containing 12.5 *μ*L* Premix Taq *(TaKaRa, Dalian, China), ROP17F (20 pmol) 0.25 *μ*L, ROP17R (20 pmol) 0.25 *μ*L, template DNA (200 ng) 2 *μ*L, and ddH_2_O 8 *μ*L, and the reaction conditions were 94°C for 5 min, then 35 cycles of 30 sec at 94°C, 30 sec at 55°C, and 1 min 20 s at 72°C, and a final extension at 72°C for 10 min. All the PCR products were then cloned into pMD18-T vector (TaKaRa, China) after purification using the DNA purification kit (TIANGEN, China) and then sequenced by Songon Biotech Co., Ltd. (Shanghai, China).

### 2.3. Sequence Analysis and Reconstruction of Phylogenetic Relationships

The* rop17* gene sequences of different* T. gondii* strains were aligned using Multiple Sequence Alignment Program, Clustal X 1.83 [18], and the sequence differences were determined according to Chilton et al. [19] and Zhao et al. [20]. Phylogenetic reconstruction was based on the* rop17* gene sequences determined in the present study plus the corresponding sequences of strains TgC7, PRU, and RH available in GenBank (accession numbers: KC997176, KC997177, and KC997178) using three inference methods, namely, neighbor-joining (NJ), maximum likelihood (ML), and maximum parsimony (MP), using the sequence of* Neospora caninum* (NCLIV_027930) as the outgroup. All the analyses were conducted following previous studies [20, 21]. Phylograms were drawn using the Tree View program version 1.65 [22].

## 3. Results and Discussion 

The length of the* rop17* genes from all examined* T. gondii* isolates was 1375 bp and A+T contents varied from 49.45% to 50.11%. The alignment of 10* rop17* sequences plus the corresponding sequences of the RH, PRU, and TgC7 strains available in GenBank revealed nucleotide polymorphisms at 33 positions, with an intraspecific variation of 0–2.1%. The genetic diversity in* rop17* gene was higher than our previous studies for PLP1 [23], ROP7 [11], eIF4A [24], and MIC13 [25] genes and the whole genome, secretome, and kinome of* T. gondii *[8]. 16 variable positions were identified as transitions and the rest variable nucleotides were classified as transversions, and no deletions were detected in the 13* rop17* gene sequences.

**Figure 1:**
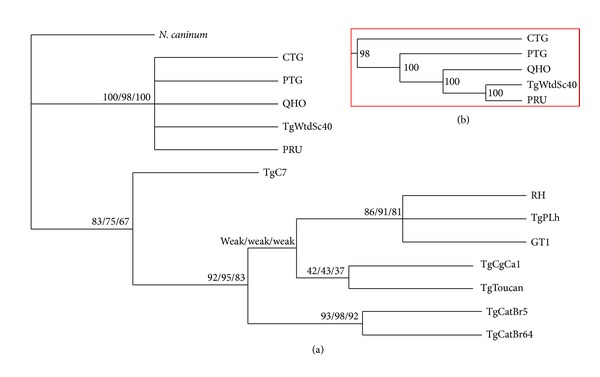
Phylogenetic analysis of 13* Toxoplasma gondii* strains based on analysis of the* rop17* gene sequences. The tree was built by neighbor-joining (NJ), maximum likelihood (ML), and maximum parsimony (MP) analyses. The numbers at notes indicate bootstrap values resulting from different analyses in the order MP/NJ/ML. (a) The much higher genetic divergence in* rop17* revealed two major clusters (denoted by I and II). (b) Subtree in the red box showing results of analysis using neighbor-joining (NJ).

Phylogeny reconstruction using MP, NJ, and ML analyses revealed two major clusters (Figure 1(a)). Topologies of all trees based on nucleotide sequences inferred by three different methods were similar, with only the small difference of bootstrap values. The classical genotypes II and III and atypical Type 12 strain were clustered in one clade. The subtree of NJ analysis further showed that genotype III (strain CTG) was separated from other strains which were supported by bootstrap analysis, and the atypical Type 12 (TgWtSc40 strain) was closely related to classical genotype II (strain PRU) (Figure 1(b)).* T. gondii* genotype II is one of the parental lineage of Type 12 based on the analysis of the inheritance of multilocus genotypes [6, 26]. The somewhat close relationship between Type II and Type 12 strains coincided with analyses of* UPRT* and* SAG1* loci [6]. All the strains belonging to genotype I in this study were clustered together, including strain TgPLh and typical strains GT1 and RH. Atypical strains TgCat1, TgToucan, TgCatBr64, and TgCatBr5 were phylogenetically clustered more closely with Type I strains. Of these, TgCatBr64 and TgCatBr5 strains which originated from cats in Brazil were grouped together. Based on the limited* T. gondii* strains examined in the present study, the* rop17* gene sequences could distinguish the major clonal lineages, but not all, showing the weak differentiation of* T. gondii* genotypes compared to analyses using GRA5, GRA6, and AK gene sequences as genetic markers [27–29]. Further validation of the* rop17* gene sequences as genetic marker is warranted by sampling more* T. gondii *strains from wider geographical locations and more hosts.

The analyses of sequence variations in nucleotides and amino acids among different strains showed high ratio of nonsynonymous to synonymous polymorphisms (>1), suggesting that* T. gondii rop17* shows signs of positive selection, although more isolates will be required to determine whether* rop17* gene is under selection at the population level. Under the immunized stresses of host cells, the positive selection occurring in* rop17* gene may increase stress resistance. Ongoing positive selection is also found in several polymorphic dense granule (GRA) antigens [30, 31] and some other ROPs [8].

## 4. Conclusion

In summary, the present study demonstrated the existence of slightly high sequence variability in the* rop17* gene sequences among* T. gondii *strains from different hosts and regions, which may be explored as a new genetic marker for population genetic studies of* T. gondii* isolates, and contributed to discovery of the new strategies for vaccination, treatment, or diagnosis.

## Figures and Tables

**Table 1:** Details of *Toxoplasma gondii *strains used in the present study.

Strain	Host	Geographical origin	Genotype∗
GT1	Goat	United States	Reference, Type I
PTG	Sheep	United States	Reference, Type II, ToxoDB number 1
CTG	Cat	United States	Reference, Type III, ToxoDB number 2
TgCatBr5	Cat	Brazil	Reference, ToxoDB number 19
TgCatBr64	Cat	Brazil	Reference, ToxoDB number 111
TgCgCa1	Cougar	Canada	Reference, ToxoDB number 66
TgToucan (TgRsCr1)	Toucan	Costa Rica	Reference, ToxoDB number 52
TgPLh	Pig	China	Type I, ToxoDB number 10
QHO	Sheep	China	Type II, ToxoDB number 1
TgWtdSc40	White-tailed deer	United States	Type 12, ToxoDB number 5

*Based on genotyping results of Zhou et al. [15, 16] and Su et al. [17].
